# Genetic Diversity of Infectious Bronchitis Virus Genotype II in Poland

**DOI:** 10.3390/pathogens15010044

**Published:** 2025-12-31

**Authors:** Anna Pikuła, Anna Lisowska, Justyna Opolska, Katarzyna Domańska-Blicharz

**Affiliations:** Department of Virology and Viral Animal Diseases, National Veterinary Research Institute, 24-100 Puławy, Poland; anna.pikula@piwet.pulawy.pl (A.P.);

**Keywords:** infectious bronchitis virus, GII genotype, virus evolution

## Abstract

The epidemiological situation in Poland for IBV GII (formerly known as D1466) has seemed stable over the years, but an increase in such infections has been recently reported. In this study, genetic characterization of the representatives of this genotype was performed in order to determine whether the new epidemic wave of GII IBV was responsible for changes in this status quo. Genotyping based on the complete S1 coding region of eight Polish IBV field strains from 2011 to 2021 confirmed that they belonged to genotype II, with two of them clustered in the two previously identified GII-1 and GII-2 lineages. In turn, the S1 coding region sequences of the next six Polish strains are very different from the previous ones and form a separate group on the phylogenetic tree. However, comprehensive analysis of all complete S1 coding regions of GII strains did not fulfill all parameters needed to create the separate GII lineage, and they all seem to belong to the GII-1 lineage. Further analysis of the partial S1 sequence of 15 IBV GII strains showed their genetic distinctiveness and indicates the ongoing evolution of this virus genotype. Considering the results of our study and the recent outbreaks of GII-2 in Western Europe, it appears that infections with GII virus strains mainly affect egg-producing, long-lived chickens, commercial layers, and breeders. Furthermore, due to the high diversity of these viruses, their circulation in the poultry population may remain undetected, and for this reason, the observed production problems in laying flocks may be attributed to other, unrelated factors.

## 1. Introduction

Infectious bronchitis virus (IBV) is one of the most influential avian pathogens affecting the poultry industry. Currently classified in the species *Gammacoronavirus galli* and *Gammacoronavirus pulli*, subgenus *Igacovirus*, genus *Gammacoronavirus*, family *Coronaviridae*, it is a virus found worldwide. The IBV genome is 27.6 kb long and encodes at least 10 open reading frames (ORFs), arranged in the 5′ to 3′ direction as UTR-1a/1ab-S-3a-3b-E-M-5a-5b-N-3′-UTR-poly(A). Four genes encode structural proteins, including the spike protein (S), envelope (E), matrix (M), and nucleocapsid (N). The S protein is transmembrane and is cleaved into two distinct polypeptides, designated S1 and S2 [1]. The S1 polypeptide determines receptor binding and is an important immunogenic component, while the S2 polypeptide is potentially a determinant of cell tropism [2,3]. In the IB virus genome, due to the lack of a proofreading mechanism of the viral polymerase, genetic mutations and recombination occur frequently. Such genetic events contributed to the remarkable antigenic and genetic diversity of the virus, which has a significant impact on disease symptoms, vaccine protection, and diagnostic methods. In particular, such changes in the hypervariable regions of the S1 coding region promote the emergence of variants and genotypes, and sometimes, if these genetic changes are in regions relevant to the immune response, they also favor the emergence of new serotypes [4,5]. Phylogenetic analysis of the S1 coding region is being used to define IBV genotypes, and eight genotypes from GI to GIX and multiple lineages within individual genotypes have currently been distinguished [6,7,8,9]. The most differentiated is the GI genotype, which contains as many as 29 lineages in total [6,10,11]. The other genotypes, GII–GVII, contain only single lineages [6]. This predominance of the GI genotype is likely to be due to its greater pathogenicity compared with other GII–GVIII genotypes, but also difficulties with diagnostics. The first representatives of the GII genotype were identified in the early 1970s in the Netherlands [12]. Over the years, they were rather rarely found [13]. Recent studies have shown that there is also a second lineage of similar viruses within this genotype [14]. In Poland, the occurrence of GII viruses was confirmed more than 10 years ago, although in subsequent years their prevalence was incidental [15,16]. In this paper, we present further studies on the occurrence and characterization of strains of this GII genotype.

## 2. Materials and Methods

### 2.1. Viruses

The study utilized 15 IBV GII strains originating from IB outbreaks between 2011 and 2021, detected during routine diagnostic examinations by the National Veterinary Research Institute (Pulawy, Poland) using the previously published rRT-PCR protocol [15]. Detailed data of the flocks studied, from which the strains originated, are given in Table 1.

### 2.2. Identification of GII-1 IBV

Viral RNA was extracted using an IndiSpin Pathogen Kit (Indical Bioscence, Leipzig, Germany) according to the manufacturer’s protocol. RT-PCR amplification of a partial S1 gene sequence spanning HVR3 was performed using the Prime Script One Step RT-PCR Kit (Takara, Kusatsu, Japan) according to the manufacturer’s protocol, using primers listed in Table 1. For full S1 coding region amplification, RT-PCR was conducted using 3 overlapping primer sets (Table 2). The purified cDNA (QIAquick Gel Extraction Kit, Hilden, Germany) were sequenced in both directions by a commercial service (Genomed, Warsaw, Poland). The complete S1 coding region was manually assembled using MEGA v12 software [17]. Sequences were only acquired if two high-quality chromatograms were obtained for each read of the amplified S1 fragment. When assembling the consensus sequence, if ambiguous bases were present, the chromatogram for each read was checked visually and the nucleotide was determined when a clear signal was visible on one of the reads.

### 2.3. Recombination Analysis

The search for recombinant sequences and crossover regions was performed using Geneconv, RDP, MaxChi, Chimera, BootScan, SiScan, 3Seq, LARD, and Phylpro, all implemented in RDP4 Beta 4.97 [18], with default settings applied. Only sequences detected by more than three methods with a *p*-value < 1 × 10^−30^ were considered as recombinants.

### 2.4. Phylogenetic Analyses

Genotyping based on the full sequence of the S1 gene of detected IBV strains was performed according to the method outlined by Valastro et al. [6]. All 199 reference strain sequences were downloaded from the GenBank database, in addition to representatives of newly described IBV genotypes, i.e., GI-28 [10], GI-29 [11], GII-2 [14], GVII-1 [7], GVIII [19], and GIX [9]. The alignment of all sequence sets was performed using the MAFFT method implemented in Geneious Prime software, 2026.0.2 (Biomatters, Auckland, New Zealand). The alignments were then exported to the MEGA program, v12.0.10. Maximum likelihood (ML) phylogenetic analyses were then conducted using the best-fitting nucleotide substitution models (GTR + G + I). Adaptive bootstrap (fast) analyses of the resultant trees were performed using a default threshold of 5.0. The pairwise distances (p-distance) between individual strains as well as between groups of strains (separated into genotypes and lineages) were calculated at the nucleotide and amino acid levels using appropriate functions in the MEGA v12. The tree visualization was performed using the MEGA v12 or the iTOL v7 online tool [20]. The phylogenetic tree accuracy was assessed with approximate-likelihood-based measures of branch supports (Shimodaira–Hasegawa (SH) approximate likelihood ratio test (aLRT)) available in the PhyML software (Montpellier, France, http://www.atgc-montpellier.fr/phyml/, accessed on 23 December 2025).

## 3. Results

### 3.1. Sequences

The study yielded seven partial (corresponding to HVR3) and eight full-length sequences of the S1 coding region of IBV GII strains circulating in Poland between 2011 and 2021. The accession numbers of the obtained IBV sequences deposited in the NCBI database are listed in Table 1.

The genetic diversity of Polish IBV GII strains illustrated by the calculated nucleotide and amino acid similarity (presented in Table 3) ranged from 89.2 to 95.9% and 83.0 to 92.0%, respectively. The earliest strain from our study, CK/PL/120/2011, showed close relatedness to representatives of GII-1 lineage, Dutch V1397 and D1466 strains, with nucleotide and amino acid similarities of 91.8–93.6% and 88–89.5%, respectively. In contrast, the CK/PL/H1145/2021 strain, detected ten years later, is more closely related to the representative GII-2 IBV. Nucleotide sequences of this Polish strain have a distance of 6.6% to Dutch D181/2018, but 10.5–11% and 8.3–10.5% to strains of the GII-1 lineage and the remaining six Polish IBVs, respectively. On the other hand, the amino acid sequence differences between the CK/PL/H1145/2021 and D181/2018 strains are 11.2% but 14.4–15.1% and 10.3–16.4% to GII-1 and six other Polish strains, respectively. The similarity of nucleotides and amino acids of six consecutive Polish strains to each other is 90.8–95.7% and 83.6–92%, respectively. In turn, the nucleotide similarity to strains of GII-1 and GII-2 is 89–93.1% and 90.7–93.4%, and deduced amino acids are 82.4–86.7% and 85.2–89.7%, respectively (Table 3).

**Figure 1:**
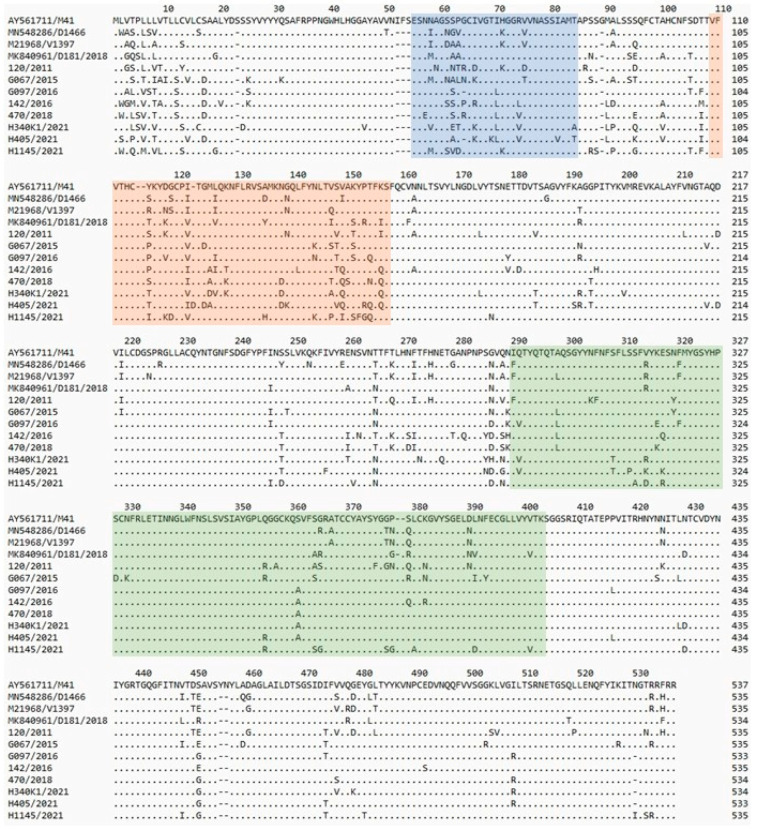
Comparison of the amino acid structure of the S1 protein of the tested and reference strains. Amino acid numbering was based on the spike-codon S1 region of IBV M41 strain. Dots show identity with reference strain sequence. The previously identified hypervariable regions of IBV-M41 of S1 [21] are highlighted in blue (HVR1), orange (HVR2), and green (HVR3).

The full-length S1 sequences of the Polish strains were 533–535 amino acids (aa) long (1599–1605 nucleotides (nt)). Compared to the three available S1 sequences of IBV genotype II reference strains (D1466, V1397 and D181/2018), a deletion of one amino acid at position 528 in four strains (CK/PL/G470/2018, CK/PL/H340/2021, CK/PL/G097/2016, and CK/PL/H405/2021), and another one at the position 94 within HVR1 of the S1 subunit in the latter two strains was observed (Figure 1). Analysis of deduced S1 amino acid sequences revealed that the Polish strains exhibit multiple amino acid changes (from 56 to 94 amino acids) compared to the reference IBV GII strains. These sequences reveal certain common aa changes characteristic of a given strain; they are most pronounced in the reference GII-1 (D1466 and V1397) and Polish CK/PL/120/2011 strains and in six Polish strains from 2015 to 2021, and least pronounced in the reference GII-2 (D181/2018) and field CK/PL/H1145/2021 strains. However, it is difficult to identify a pattern of common amino acid modifications that could be considered to be the ‘fingerprints’ of each of these virus groups.

### 3.2. Phylogenetic Analysis

**Figure 2:**
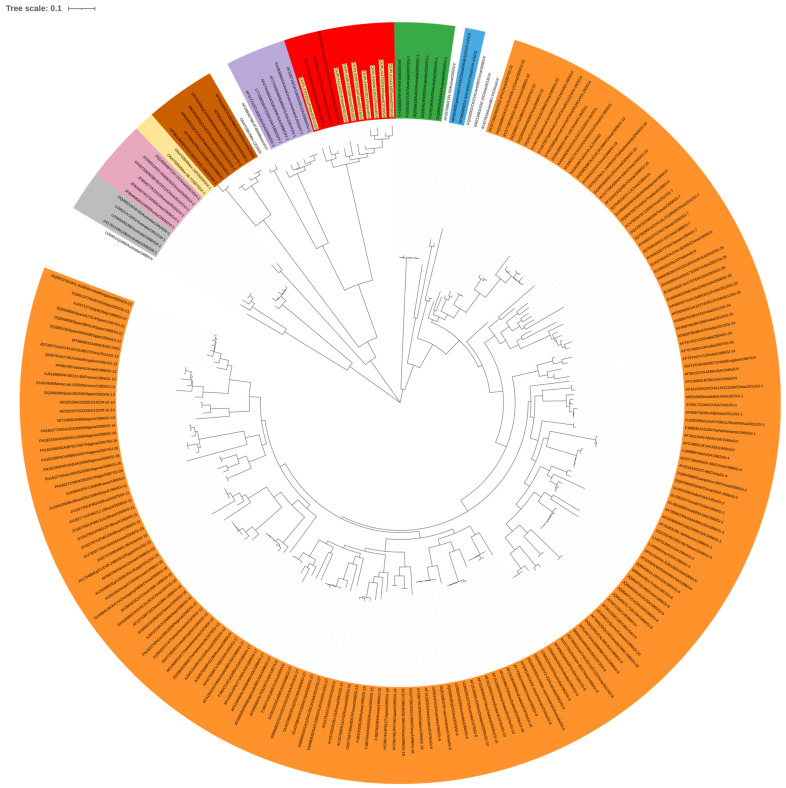
Maximum likelihood phylogenetic analysis of the S1 sequence of Polish IBV strains ac-cording to Valastro et al.’s rules [6]. The tree was constructed using the GTR + G + I model with adaptive bootstrap (default threshold of 5.0.) iterations. The main IBV genotypes (GI–GIX) are denoted with designations and colors: GI—orange, GII—red, GIII—grey, GIV—purple, GV—green, GVI—pink, GVII—blue, GVIII—brown, GIX—yellow; the taxon names of the Polish strains characterized in the presented study are highlighted in yellow.

**Figure 3:**
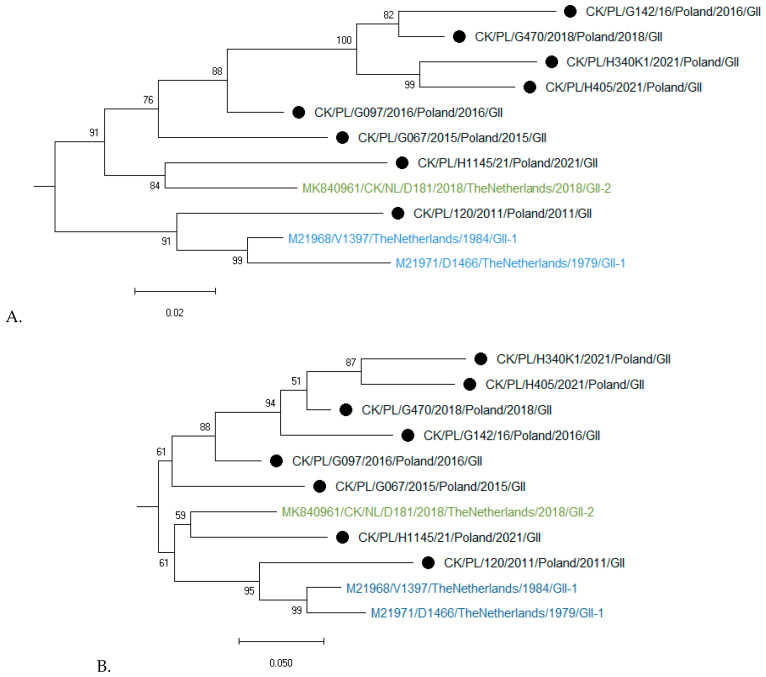
Maximum likelihood phylogenetic analysis of GII IBV strains of the S1 nucleotide (**A**) and amino acid (**B**) sequences. Trees were constructed using adaptive bootstrap iterations and GTR + G + I and WAG + I + F models for nt and aa sequences, respectively. The taxon name of the Polish strains characterized in the presented study is marked with a black dot. Names of reference strains are in blue or green colors.

Genotyping based on the complete S1 coding region confirmed that all eight studied strains represent genotype GII of IBV (Figure 2). Phylogenetic analysis based on nucleotide (nt) and amino acid sequences of Polish and reference GII strains grouped them into three clusters (Figure 3A,B). CK/PL/H1145/2021 was grouped with a representative of GII-2, the CK/NL/D181/2018 strain. In turn, the strain CK/PL/G120/2011 formed a common branch with reference strains D1466 and V1397, historically classified as GII-1, and calculated SH-aLRT values of these clusters were 1 and 0.98, respectively. The remaining Polish IBVs located in the third cluster had an SH-aLRT value of 0.97. The pairwise distances of GII-1 viruses were 3.8–10.8% and 5.7–13.9% for nucleotide and amino acid sequences, respectively. Similarly, the values of this parameter for IBV clustering with historic GII-2 were 6.7% and 10.8%. For GII-3 viruses the P-distance values were 4.1–10.8% and 8.4–15.7% for nucleotide and amino acid sequences, respectively. In turn, the pairwise distances of GII and GI–GIX genotypes ranged from 37 to 42% and from 37 to 49% for nucleotide and amino acid sequences, respectively. As numerous individual distance values overlap between identified clusters, and calculated SH-aLRT values for them do not reach the recommended value of >0.98, the comparison of ‘between-group mean genetic distances’ was additionally performed. The pairwise distances of GII-1, GII-2, and the third group presumed from the phylogenetic trees were 9.3–10.3% and 13.7–15.2% for nucleotide and amino acid sequences, respectively (Supplementary Tables S1–S4).

**Figure 4:**
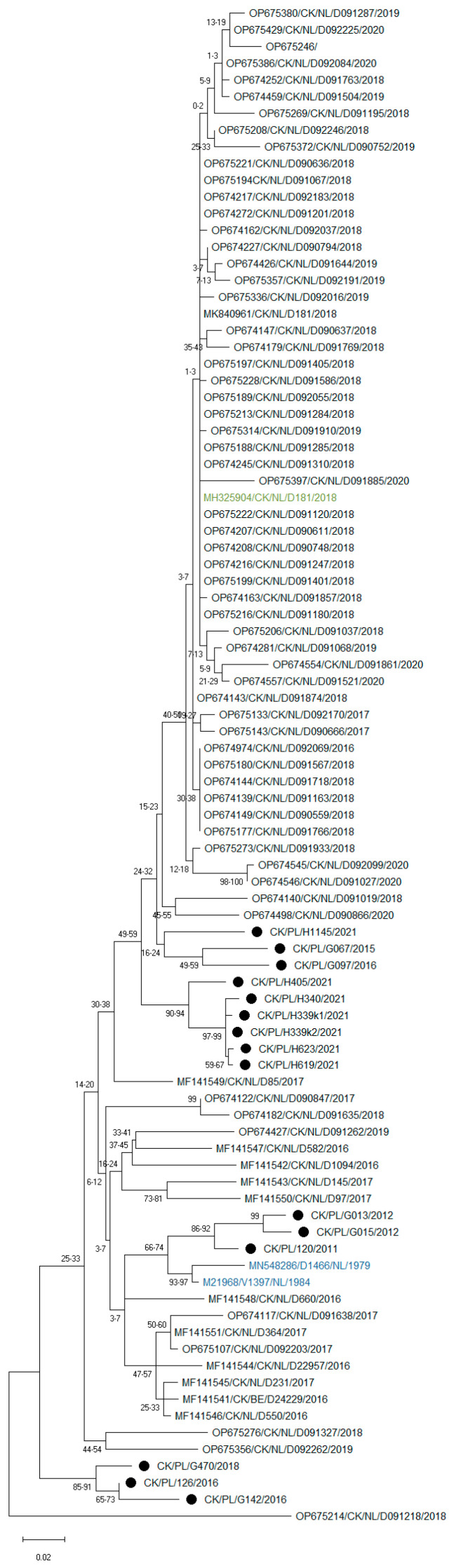
Maximum likelihood phylogenetic inference of GII IBV strains based on nucleotide sequences of the HVR3 region of the S1 gene. The tree was constructed using 1000 bootstrap iterations and the TIM3 + F + I + G4 model. The taxon name of the Polish strains is marked with a black dot. Names of reference strains are in blue or green colors.

Attempts to obtain the full S1 sequence for all 15 tested strains were unsuccessful; however, a sequence of the HVR3 region of the S1 gene was obtained, which is commonly used for IBV diagnosis. As dozens of such S1 IBV GII sequences have recently been deposited in the NCBI database, a phylogenetic analysis was also performed for this shorter S1 fragment. The results of phylogeny also showed high variation among the studied strains (Figure 4). Interestingly, most of the Polish IBVs formed independent branches on the phylogenetic tree, except for three strains from 2011 to 2012, which clustered with GII-1 reference strains, confirming the high genetic diversity of circulating GII strains in Poland.

Recombination analysis for the criteria used did not confirm the presence of recombinants.

## 4. Discussion

D1466-like IBVs first emerged in the Netherlands in the late 1970s [12]. At that time, an increased number of flocks suffering from problems with egg production was reported. Further characterization of these strains revealed that they are antigenically different from known IBV strains, and the available vaccines do not provide protection against disease symptoms resulting from their infection. In later years, the spread of the virus in Europe was demonstrated, but its prevalence remained low [13]. Nevertheless, this epidemiological landscape has recently changed, as the next wave of D1466-like IBV infections in layers and breeders has been reported in the Netherlands, Belgium, and Germany [14,22]. Studies have shown that these strains represent the new lineage within genotype II (GII-2). Furthermore, a cross-neutralization test confirmed that strain D181/2018 represents a new IBV serotype. In Poland, the first case of D1466-like IBV infection was confirmed at the end of 2011, and the virus subsequently spread across the country, with a prevalence of 11.7% [16]. In subsequent years, these strains were sporadically detected; more recently, an increase in GII IBV infections has been observed, raising questions about the reason for this change. Hence, taking into account the recently reported IB epidemic caused by the new lineage GII-2, a study was undertaken to molecularly characterize the genotype II strains circulating in Poland over the past decade.

A comprehensive analysis of the full sequence of the S1 coding region of strains from 2011 to 2021 suggests that, despite considerable diversity, the identified Polish GII IBV strains belong to a single lineage. According to the IBV classification rules proposed in 2016, several criteria must be fulfilled for a given strain to form a new lineage. These include the formation of a monophyletic group of at least three viruses collected from at least two different outbreaks, with strongly supported nodes (>0.98 SH-like test value recommended) and a pairwise distance not exceeding 13% for nucleotide sequences and 14% for amino acid sequences. The IBV classification should not be based solely on one of these criteria but requires all data to be taken into account [6]. The earliest strain, CK/PL/120/2011, showed close relatedness to representatives of the GII-1 lineage, specifically Dutch strains V1397 and D1466, with nucleotide and amino acid pairwise distances of 3.8–10.8% and 5.7–13.9%, respectively. In contrast, strain CK/PL/H1145/2021, detected ten years later, is more closely related to the reference Dutch D181 strain, identified in 2018, which was stated as being of GII-2 linage [14], and their p-distance values were 6.7 and 10.8% for nucleotide and amino acids, respectively. Six consecutive Polish strains belonged to the third cluster, and their nucleotide and amino acid pairwise distances were 4.1–10.8% and 8.4–15.7%, respectively. The above data indicates that many of these individual distance values overlap between identified clusters, which limits the interpretative power of this approach. Moreover, although the topology of phylogenetic trees indicates the existence of three clusters, they are not sufficiently supported statistically, as the calculated SH-aLRT values do not exceed the recommended threshold of 0.98. To strengthen the evolutionary and taxonomic interpretation, between-group mean genetic distances were calculated, which allow direct and statistically meaningful comparisons between lineages. The results obtained were surprising: between-group mean genetic distances for the probable GII-1, GII-2, and GII-3 clusters were lower than the calculated values for separate lineages within the GI genotype. The lowest mean genetic distance values were found between the GI-22 and GI-29, and between the GI-19 and GI-28 lineages, at 12.5% and 13.5%, and 14.5% and 12.2% for nucleotide and amino acids, respectively. The differences between the presumed GII-1, GII-2, and GII-3 lineages ranged from 9.3 to 10.2% for nucleotides, and from 13.7 to 15.2% for amino acids. Furthermore, if we assign Polish strains to the GII-2 group in such a comparison, the mean genetic distance values between them were 10.1% and 14.9% for nucleotide and amino acids, respectively. The remaining mean genetic distance values between lineages within the GI genotype are mostly above 20% for both levels. Considering the overall diversity of the S1 coding region among lineages within the GI genotype, it seems that all GII strains described to date belong to a single lineage. Perhaps the characterization of subsequent strains will enable a more unambiguous classification of these strains to subsequent GII lineages.

In this study, eight different primers were constructed and, despite using them in various combinations, the full sequence of the S1 coding region required for accurate IBV classification could only be determined for eight of the fifteen strains detected. This is most likely due to the large variation in the nucleotide composition of this structure among the viruses studied. The public nucleotide database GenBank contains many sequences of GII strains from Western European countries, most of which are only a short HVR3 fragment of the S1 coding region. As is shown in Figure 4, there is considerable variation in this fragment between strains, and Polish strains are grouped separately. However, to determine if these European strains constitute further lineages of GII, the sequence of the entire S1 coding region is required, and as mentioned above, this is a very tedious task.

The comprehensive analysis presented above provides insight into the evolutionary relationships between the strains studied, but it does not provide answers about their origin. A large number of substitutions and even deletions can be the cause of viral fast evolution due to the error-prone nature of RNA-dependent RNA polymerase, genomic architecture, and replication speed [23]. The identified high genetic diversity between strains is not the effect of recombination on the structure of the S1 protein, as conducted analysis using the RDP4 software did not confirm such events among Polish strains. However, such high S1 variability identified in Polish GII strains may be surprising, as their evolution took place in the absence of environmental pressure, i.e., without the use of homologous vaccines. The impact of applied vaccinations on the diversity of strains circulating in the field has been recognized as significant; however, opinions on this subject have varied. Cavanagh et al. estimated the GI-13 IBV mutation rate in the absence of vaccination pressure to be 3 × 10^−3^ substitutions/site/year [24]. Whereas, another study showed that GA98 strains (GIV) had a higher mutation rate (1.5 × 10^−2^ s/s/y), which was attributed to vaccination pressure [25]. In turn, the study of Flageul et al. [26] revealed that IBV of the same GI genotype but of a different lineage than the vaccine strain evolved quite differently in vaccinated and unvaccinated chickens. A reduction in viral diversity was observed in vaccinated birds, most likely through a reduction in viral RNA load as a result of vaccine-induced immunity. At this time, it is unknown whether a similar effect would occur with GI-based vaccines acting on GII strains. On the other hand, further studies have shown that IBV variability in an adapted host (chicken) does not stabilize even without immune pressure, generating continuous molecular changes in its genome. The Shannon entropy values measured during IBV GI-23 passages in SPF chickens varied for numerous genes, including the S gene, which had the highest complexity and was subjected to positive selection [27]. It seems that GII genotype viruses may undergo similar evolutionary processes. However, given the limited number of isolates, problems with accurate molecular characterization resulting from the high diversity of these strains, infrequent sampling over time, and their wide geographical distribution, it cannot be ruled out that the identified S1 variability of GII strains in Poland is the result of multiple introductions of these strains into the country. It is not known whether similar GII strains are more widespread in other European countries or even outside of Europe. The lack of reports on their wider occurrence may result from rather problematic diagnostics (which are labor-intensive and time consuming), but also from their relatively low pathogenicity.

The results of our study also show that knowledge of the prevalence of infection with IBV GII strains is very limited, which is undoubtedly related, firstly, to laborious diagnosis and, secondly, to their rather low pathogenicity. Molenaar et al. assessed in silico the suitability of universal primers used for IBV detection and differentiation of D1466-like strains [14], and the results showed that most of them may fail to detect GII-2 due to the high number of nucleotide mismatches. Our comparisons also led to similar conclusions regarding the detection of the new lineage. To date, IBV GII has been exclusively identified in Europe; moreover, this genotype shows a large evolutionary distance compared to other IBVs [6]. Perhaps this is one of the reasons for the low detection rate of such strains in routine laboratory diagnostics. It is not negligible that the strains show relatively low virulence. IBV strains of genotype II mainly affect layers and breeders, and cause impairment of the reproductive system, mainly resulting in a decrease in egg production [14,28]. Unfortunately, there is no information on the observed disease symptoms in studied flocks, but interestingly, all cases have been confirmed in layers and breeders that have already started egg production (Table 1). Moreover, poorly expressed disease symptoms may favor the spread of these strains, which is further supported by low vaccine protection due to the lack of homologous vaccination.

## 5. Conclusions

In conclusion, IB viruses of genotype GII have circulated in the poultry population in Poland over the last decade. The identified viruses have a highly diverse S1 coding region. Some of these viruses are very similar to the previously known GII-1 and 2 lineages, but most of them form a separate group on the phylogenetic tree, indicating the continuous evolution of this virus genotype. However, based on a comprehensive analysis of these strains and a comparison with the diversity of lineages in the GI genotype, it cannot be concluded with certainty that they form a separate lineage. Considering the results of our study and the recent outbreak of GII in Western Europe, it appears that such infections mainly affect egg-producing, long-lived chickens, commercial layers, and breeders. Due to the high diversity of these viruses, they may circulate undiagnosed.

## Figures and Tables

**Table 1:** List of field IBV GII strains circulating in Poland 2011–2021.

Strain	Collection Date	Chicken		Localization (Voivodeship)	GenBank Accession No.
		Age	Type		
G120/2011	2011/11	30 w	Layer	Lubusz	OR437306 *
G013/2012	2012/01	35 w	Broiler breeder	Greater Poland	OR437299
G015/2012	2012/01	49 w	Broiler breeder	Greater Poland	OR437300
G067/2015	2015/05	ND	ND	Greater Poland	OR437307 *
G097/2016	2016/03	35 w	Breeder	Masovia	OR437308 *
G126/2016	2016/05	29 w	Layer	Silesia	OR437301
G142/2016	2016/05	31 w	Breeder	Warmia-Masauria	OR437309 *
G470/2018	2018/10	29 w	Breeder	Masovia	OR437310 *
H339K1/2021	2021/03	43 w	Breeder	Masovia	OR437302
H339K2/2021	2021/03	43 w	Breeder	Masovia	OR437303
H340/2021	2021/03	43 w	Breeder	Masovia	OR437311 *
H405/2021	2021/03	91 w	Layer	Masovia	OR437312 *
H619/2021	2021/04	44 w	Breeder	Masovia	OR437304
H623/2021	2021/04	41 w	Breeder	Masovia	OR437305
H1145/2021	2021/05	2–24 m	Layer backyard	Łódź	OR437313 *

w—week, m—month, ND—no data, * full-coding sequence of S1.

**Table 2:** Primer sequences used in the study for the amplification of a partial and full sequence of S1 gene.

Primer	Sequence (5′ ⟶ 3′)	nt Position vs. D1466	Size of Amplicon
Partial S1			
HVR3F	CCCTAAAGGTAGGTTAGCATG	666–689 ^1^	498 bp
HVR3R	TCYCCAWTATAAACACCCTTACA	1141–1163 ^1^	
Full-coding S1			
100S-F	GAGTTGAAAYTAAAAGCAACGC	19,714–19,735 ^2^	704 bp
607S-R	CAGTGTCCTTCATAATTCTACG	586–607 ^1^	
310S-F	GTGTTTGTAACCCATTGTGGG	310–330 ^1^	853 bp
HVR3R	TCYCCAWTATAAACACCCTTACA	1141–1163 ^1^	
1031S-F	GTGTTTCTATTAGCTATGGACCA	1031–1053 ^1^	716 bp
1747S-R	GCACGTAATCAGTCCTATTAAG	1726–1747 ^1^	

^1^—spike; ^2^—polyprotein 1ab.

**Table 3:** Nucleotide and amino acid similarity of the S1 gene of genotype II IBDV strains was calculated using Geneious Prime software.

IBV Strain	D1466	V1397	D181/2018	120/2011	G067/2015	G097/2016	G142/16	G470/2018	H340/2021	H405/2021	H1145/21
**D1466**		94.2	86.2	88.0	83.9	85.6	85.2	85.6	83.6	82.4	84.9
**V1397**	96.3		88.2	89.5	85.6	87.5	86.2	86.7	85.6	83.7	85.6
**D181/2018**	90.3	92.0		85.6	87.3	88.8	84.9	86.9	87.1	85.2	88.8
**120/2011**	91.8	93.6	90.6		84.3	84.9	83.7	84.3	83.2	83.0	83.7
**G067/2015**	89.9	91.7	93.1	90.5		89.0	85.2	85.2	83.6	84.9	86.4
**G097/2016**	89.9	92.1	92.9	90.2	94.2		89.5	91.6	88.4	90.1	87.9
**G142/2016**	90.0	92.0	90.7	90.1	91.7	93.3		92.0	86.5	87.3	83.6
**G470/2018**	90.0	91.8	91.2	90.0	91.7	94.8	95.9		90.8	90.8	85.4
**H340K1/2021**	89.3	91.3	91.4	89.2	90.8	93.2	93.2	95.4		90.3	85.2
**H405/2021**	89.2	90.9	90.7	89.4	91.5	94.2	93.3	95.3	95.7		89.7
**H1145/2021**	89.0	90.5	93.4	89.8	92.1	91.7	89.5	89.8	89.6	89.7	

## Data Availability

The obtained genome sequences generated in this study were submitted to the GenBank database (https://www.ncbi.nlm.nih.gov/genbank/, accessed on 23 November 2025) under accession numbers OR437299–OR437313. The original contributions presented in this study are included in the article/Supplementary Materials. Further inquiries can be directed to the corresponding author.

## Supplementary Materials

The following supporting information can be downloaded at: https://www.mdpi.com/article/10.3390/pathogens15010044/s1, Table S1. The pairwise distances (p-distance) between individual strains were calculated at the nucleotide level using appropriate functions in MEGA v12. The obtained values for Polish strains characterized in the presented study are yellow-highlighted [29,30]; Table S2. The pairwise distances (p-distance) between individual strains were calculated at the amino acid level using appropriate functions in MEGA v12. The obtained values for Polish strains characterized in the presented study are yellow-highlighted [30]; Table S3. The pairwise distances (p-distance) between groups of strains (separated into genotypes and lineages) were calculated at the nucleotide level using appropriate functions in MEGA v12. The obtained values for Polish strains characterized in the presented study are yellow-highlighted. The lowest values between the given lineages of GI are bolded and grey-highlighted [29,30]; Table S4. The pairwise distances (p-distance) between groups of strains (separated into genotypes and lineages) were calculated at the amino acid level using appropriate functions in MEGA v12. The obtained values for Polish strains characterized in the presented study are yellow-highlighted. The lowest values between the given lineages of GI are bolded and grey-highlighted [30].
